# Supplementary material for: Factors associated with knowledge and practice regarding oxygen administration: A cross-sectional study among registered nurses working in wards and ICUs at Muhimbili National Hospital in Dar es Salaam, Tanzania
Source: PLoS One. 2025 Jan 16;20(1):e0317620. doi: 10.1371/journal.pone.0317620 (PMC11737716; doi:10.1371/journal.pone.0317620)
Supplement: S1 Appendix — (PDF) [file pone.0317620.s001.pdf]

S1 Appendix: Items assessed in the questionnaire covering knowledge, practices, and organizational factors affecting oxygen administration

Questions regarding knowledge of oxygen administration

1. What is the general consideration of O2 administration?
   a) Oil or grease around O2 connection should be avoided
   b) Alcohol, ether, and other inflammatory liquid should be used with caution in the vicinity of oxygen
   c) Smoking in the vicinity of oxygen
   d) Oxygen cylinders should be kept secure position
2. What is the indication of oxygen administration?
   a) Hypoxemia
   b) Increased work of breathing
   c) Increased myocardial word
   d) Pulmonary hyperventilation
3. What are the precaution during administering oxygen therapy?
   e) Carefully assessing its effects on each patient
   a) Oxygen in medication
   b) Prescribed by physician
   c) The nurse assesses the patient frequently for confusion, restlessness progressing to lethargy, diaphoresis, pallor, tachycardia
4. What is the contraindication to oxygen administration?
   a) No absolute contraindication
   b) Chronic carbon dioxide retention
   c) Related to the danger of hyperoxemia
   d) Fire hazard
5. Selection of an appropriate oxygen delivery device must be
   a) based on PaO2
   b) based on guideline
   c) based on doctor order
   d) patent condition
6. oxygen must be only administered at the
   a) rate
   b) percentage
   c) level
7. Approximated oxygen concentration delivered by nasal cannula
   a) 22 -24 %
   b) 26 -28 %
   c) 32 -36 %
   d) 36 -40 %
   e) 40 -44 %
8. What is the nursing care for a patient receiving oxygen by nasal cannula?
   a) keep nosepieces clean
   b) evaluate for presser sore over ears, cheeks, and noses
   c) lubricate nasal prongs
9. What is the problem associated with simple face mask?
   a) the mask needs to be removed for eating and drinking
   b) tight seal can cause facial irritation
   c) can feel hot
   d) cause anxiety in some people (child)
10. What are the signs and symptoms of oxygen toxicity
   a) Coughing, chest pain, trouble breathing, and blue vision
   b) Coughing, chest pain, edema, and blue vision
   c) Coughing, chest pain, breath, and blue vision
   d) Coughing, chest pain, lung, and blue vision
11. What is the medical treatment of oxygen toxicity
   a) Reduced exposure to oxygen and exogenous antioxidant
   b) Increasing exposure to oxygen and exogenous antioxidant
   c) Reduced exposure to exogenous antioxidant

Questions regarding Oxygen administration practices

Practices Before oxygen administration:

1. Verify physician prescription before administration:
2. Wash hands
3. Prepare needed equipment
4. Introduce yourself to patient
5. Identify the patient
6. Explain procedure to the patient
7. Document baseline observation including saturation
8. Is pulse oximetry available to monitor response to oxygen therapy
9. Disinfect hand
10. Wear disposable gloves

Practices During oxygen administration:

11. Do you assess patient oxygen saturation
12. Do you assess patient respiratory status for the normal and abnormal finding
13. Connect the flow meter to the oxygen supply
14. Fill the humidifier with a suitable amount of distilled water
15. Open oxygen supply before connecting oxygen device to the patient
16. Connect the oxygen device to the oxygen setup with humidification
17. Adjust flow rate of oxygen according to the prescribed rate
18. Connect oxygen therapy devices to the patient appropriately
19. Connect tubing over and behind each ear with an adjuster comfortable to the patient
20. Placing tubing around the patient's head with the adjuster at the back or base of the head
21. Place gauze pads at the ear beneath the tubing if necessary
22. Adjust the fit of the device tubing to make the patient feel comfort
23. Reassess the patient's respiratory status

Practices after oxygen administration:

24. Discard used equipment
25. Remove gloves
26. Document date and time of connecting oxygen therapy
27. Assess the patient's condition before and after the intervention to assess any improvement in the patient's status.

Questions regarding organizational factors

1. Lack of training courses
2. Unavailability of equipment and supplies
3. Lack of periodic maintenance of equipment/devices
4. Unavailability of a standardized protocol for oxygen therapy
5. Unclear and incomplete written prescription for oxygen therapy
6. Oral prescription to oxygen therapy only
