# Supplementary material for: Factors associated with knowledge and practice regarding oxygen administration: A cross-sectional study among registered nurses working in wards and ICUs at Muhimbili National Hospital in Dar es Salaam, Tanzania
Source: PLoS One. 2025 Jan 16;20(1):e0317620. doi: 10.1371/journal.pone.0317620 (PMC11737716; doi:10.1371/journal.pone.0317620)
Supplement: Attachment — Submitted filename: PLOS ONE Comments..11.docx [file pone.0317620.s002.docx]

**Manuscript no: PONE-D-23-37459**

Title: Factors associated with knowledge and practice regarding oxygen administration: A cross-sectional study among registered nurses in Dar es Salaam, Tanzania

I would like say thank you for giving the chance to review this manuscript.

**Title**

It is a good title that assess the HCWs in the health facilities and support the different stakeholders.

Generally, it is good. However, the following comments/questions need to be considered before publication of this manuscript.

**Abstracts**

You include all sub-section of abstract, which is good. It is better if you remove less important finding from results subsection, for better attraction of readers.

-‘’ Of the 208 respondents, 120(57.7%) were female, 144(69.2%) were in the age group of 20-40, and 175(84.1%) worked in the wards”

- ‘’ None of the socio-demographic (including knowledge of oxygen administration)

and organizational factors were associated with practice on oxygen administration”

**Materials and Methods**

- You start with study design. It is better if begin from study setting since most of the articles are like that.

- You didn’t mention your study period.

- You specifically mention study population, again it is advisable to replace by population/participants characteristics since it include study criteria.

- Under data management and analysis, which type of SPSS Version you have been used specifically? You wrote two version, be consistent. 23/25?

- What cut of point you have been used during bivariate logistic regression to select the candidate of multivariate ?

- You didn’t mention nothing about your data entry mechanism? Model fitness?

**Results**

- What was the mean age of your respondents ?

- Table 3? It is not clear for the readers? Try to modify as per standard of regression table. Knowledge status (Good vs Poor; No of/frequency of single among good knowledge vs poor knowledge)? How some body know whether your COR calculation is correct or not? It is better if you modify it.

- Table 4 ? How you identify candidate variables ? by p-value <0.2/from previous articles/scientific consideration ? Did you consider the reason why not statistically significant but clinically significant ?

**Discussion**

- What is your standard reference to say lower or higher proportion ? I mean you didn’t incorporate CI for your both knowledge and practice ? Include it.
