# Supplementary material for: Factors associated with knowledge and practice regarding oxygen administration: A cross-sectional study among registered nurses working in wards and ICUs at Muhimbili National Hospital in Dar es Salaam, Tanzania
Source: PLoS One. 2025 Jan 16;20(1):e0317620. doi: 10.1371/journal.pone.0317620 (PMC11737716; doi:10.1371/journal.pone.0317620)
Supplement: Attachment — Submitted filename: Response to Reviewers.docx [file pone.0317620.s003.docx]

**Factors associated with knowledge and practice regarding oxygen administration: A cross-sectional study among registered nurses in Dar es Salaam, Tanzania**

**Response to reviewer’s comments**

Again, we thank the reviewer for the comments provided to improve this study. We have revised the manuscript as shown in the point-to-point responses to reviewer’s comments provided in the table below. If any of our responses do not adequately address the comments, please let us know.

| **Additional Editor Comments:** | **Authors Response** |
| --- | --- |
| **Abstract:** |  |
| Limit the amount of information in the background section of the abstract. On the other hand, the method section misses key information such as the study period, tool, and method of data entry. | We appreciate the comment. The information in the background section of the abstract has been reduced. The study period, data collection tool, and method of data entry have been added in the methods section |
| In the result section, remove unnecessary detail in reporting the background characteristics of the participants. Include the 95% CI when you report the outcome variables. Remove either the p-value or the CI from the factors; it transmits the same message. | Thank you for this comment. Unnecessary detail in reporting the background characteristics of the participants has been removed from the result section. We have also removed the CI from outcome factors to remain with p-values only |
| **Introduction:** |  |
| A paragraph must describe a single idea; the breakdown paragraph is at line 51. A detailed explanation of hypoxia and hypoxemia is not necessary. Above all, the background lacks the proportion of knowledge and practice among nurses from the previous studies. Address the factors associated with knowledge and practice separately. | We appreciate he comment. We have revised the paragraphs to carry one idea and have removed unnecessary details regarding hypoxia and hypoxemia. We have added the proportion of knowledge and practice among nurses from the previous studies and addressed the factors associated with knowledge and practice separately. |
| Support the descriptions with figures; for example, in line 73, you write, “In Tanzania, poor administration of oxygen therapy is a growing problem." What is your evidence? Are there any proposed solutions so far? | We appreciate the comment. We have revised to generally cover challenges experienced in low-income settings with regard to oxygen administration |
| **Methods and materials:** |  |
| Modify the order of the subsections as suggested by one of the reviewers. The study setting must be written in relation to your title. Since your study is on nurses, you have to mention the total number of nurses working in the hospital. | We appreciate the comment. The order of subsections has been modified to begin with study settings. Muhimbili National Hospital has been added to the title as the setting of the study. The total number of nurses working in the hospital has been mentioned in the study setting |
| Exclusion criteria: you exclude those nurses with less than one year of experience caring for patients in need of oxygen therapy. On the other hand, experience is considered a variable. How? | We appreciate the comment. This was an oversight. We have removed this criterion since nurses with less than one year of experience were part of the study |
| Why the correction formula? It is better if you include all of them. Some of the problems observed in the model are due to the small sample size. | We appreciate the comment. We have revisited the subsection -containing this information and revised information regarding the used formula |
| The approach used to select the participants is not scientific. Why do you not use a table of random numbers in Excel by using the nurse's ID? Did you really write on 360 pieces of paper—208 yes and no for the rest???? | We appreciate this important observation. However, we did not use a table of random numbers in Excel by using the nurse's ID. We had two pieces of paper written YES and NO, folded and put in the box. A nurse was asked to pick one, those who picked YES paper were included in the study. We have made a revision to clarify this.  The confusion stemmed from the information we included in the sample size and sampling procedure subsection which was based on the data we were provided with during development of the proposal and before the actual data collection was done. We have corrected this error by removing the information that was presented in the table from this subsection and made revisions based on the actual information obtained during data collection. |
| Modify the subheadings under the method section; e.g., “Replace “’Data collection’’ with ‘’data collection procedure’’ | We thank you for the comment. We have replaced “’Data collection’’ with ‘’data collection procedure’’ |
| How did you minimize bias during the data collection? | We appreciate the comment. To minimize bias, first, anonymity and confidentiality were assured to respondents to reduce social desirability bias, where respondents may provide answers they think are socially acceptable rather than truthful. Second, we performed data cleaning and validation checks to identify and address any inconsistencies or errors in the data. These have been described in the data collection subsection. We have also added information regarding data cleaning and validation checks in the data management and analysis subsection. |
| Nothing is written about the software used for the data entry. Which version of SPSS was used? 23 vs. 25. Be consistent. The criterion to include variables for the multiple logistic regression must be mentioned here. | We thank you for the comment. We agree and have made revisions. We have added information about software used for analysis i.e. the IBM Statistical Package for Social Sciences (SPSS) version 25. Variables from the bivariate analysis which were included in the multiple logistic regression were those with p values less than 0.2. |
| **Result:** |  |
| Table 2: Why knowledge and practice are presented with the socio-demographic. | Thank you for the comment. We have removed knowledge and practice from the table of sociodemographic characteristics |
| Table 3. Add the cross-tabulation for each variable (the poor category must be included). | We have added the cross-tabulation for each variable (including poor category that was missing) |
| Moreover, the operational definition of good vs. poor knowledge is not clear. Under the method section, you write that the mean is used as a cutoff point. In contrast, in this table, you put proportion. How? The mean you put in lines 202 is not clear. Knowledge was assessed by 11 items; how is the mean 60? | We appreciate the comment. We have revised the cut-off point used in the tables by removing the proportions and replaced with the mean to reflect what is described as cut-off point in the methods section. The mean for knowledge has been corrected to be 6.6(2.1) |
| Table 4. Run the multiple logistic regression even if there is no significant variable in the bivariable analysis. | We appreciate the comment. We have run the multiple logistic regression even if there is no significant variable in the bivariable analysis. |
| **Discussion:** |  |
| The 95% confidence interval is mandatory to compare your finding with the previous studies. | We appreciate the comment. We have taken into consideration the 95% confidence interval to compare our finding with the previous studies. |
| The justifications given for the discrepancies are very shallow and not supported by evidence. | We appreciate the comment. We have revised the justification for discrepancies and supported them with evidence |
| Add the clinical implications and scientific contribution of this study. | We appreciate the comment. We have added the clinical implications and scientific contribution of our study |
| In general, the manuscript needs further reanalysis and revision of all its components | We appreciate the comment. We have done our best to reanalyze and make revisions in all sections |
|  |  |
| **Reviewer #1** |  |
| General comment:  Overall, the manuscript is well written, and it addresses important issue “Knowledge and practice regarding oxygen administration” in low-resource countries like Tanzania. The problem has been well justified with the support of extensive literature review. The methods and results are well presented. However, I have few comments that would help to further improve the manuscript: | We appreciate the comment |
| **Major comments** |  |
| 1. Abstract: Briefly describe the measurement of outcome variable in the abstract section of the manuscript | We agree with the comment. We have briefly described the measurement of outcome variable in the abstract section of the manuscript |
| 2. Validity and reliability: I recommend reporting the statistical measures of reliability for Likert scale questions (e.g. Cronbach alpha) | We agree with the comment. We have included the statistical measures of reliability for Likert scale questions (measuring oxygen administration practice) using Cronbach alpha in the results section |
| 3. Multiple logistic regression: I would recommend setting the criteria to select variables to include in the logistic regression model and describing it in the methods section. My thinking is that the model would have been improved by excluding sex and marital status because their p-values are very far from 0.05. | We agree with the comment. We have used the criterion of p-values ≤ 0.2 to select variables to include in the logistic regression model and have described it in the methods section. |
| **Minor comments:** |  |
| 4. Line 27: Consider rephrasing “A p-value of less than 0.05 was used to determine significant relationships” to “the p-value less than 0.05 was considered statistically significant”. This is because p-value less than 0.05 is just a criterion but not statistical method itself. The method used here was logistic regression. | We agree with the comment. We have changed the sentence “A p-value of less than 0.05 was used to determine significant relationships” to read “Statistical significance was determined at a p-value less than 0.05.” |
| 5. Line 37 and line 324: I would recommend interpreting proportion instead of absolute number in the conclusion. | We agree with the comment. We have revised the statement to read ‘’a substantial proportion…” |
| 6. Line 39-541: The statement “Further research is needed to determine the relationship between length of work experience and knowledge of oxygen administration” Contradict with the presented results where authors report that experience was significantly associated with knowledge of oxygen administration. Consider rephrasing it. | We agree with the comment. We have removed the statement since the results have changed, i.e. the relationship between length of work experience and knowledge of oxygen administration is no longer statistically significant after re-analysis |
| 7. Line 82: Rephrase “A descriptive, cross-sectional …” to “ A descriptive cross-sectional…”. I think Comma (,) is misplaced between the term “descriptive” and “cross-sectional”. | We agree with the comment. We have removed the comma between the term “descriptive” and “cross-sectional”. |
| 8. Line 92: “According to the hospital website”. The website should be appropriately cited as per recommended journal style. | We agree with the comment. We have revised and cited the hospital website as according to recommended journal style |
| 9. Line 107: Consider rephrasing the statement “A sample size was calculated…” to “a sample size was estimated using the cross-sectional formula for study when the population size is known”. | We agree with the comment. We have rephrased the statement to read “A sample size was estimated using the cross-sectional formula for study when the population size is known” |
| 10. Line 108: For readability purpose, the formula should not be presented inside the paragraph containing texts. | We agree with the comment. We have removed the formula from the text |
| 11. Line 110: I would recommend motivating the use of 10% as a non-response rate. Was it based on literature or something else? | We agree with the comment. It is a practice that emerges from methodological discussions and practical considerations in research. We have decided to remove it. |
| 12. Line 136-148 (Measurements): I would recommend you organize into paragraphs (if possible, use sub-titles). One or two paragraphs for of outcome variables (knowledge, practice) and one paragraph for explanatory variables (demographics, organizational, etc). | We agree with the comment. We have organized measurements one paragraph of explanatory variables and two paragraphs od outcome variables. |
| 13. Line 173: the version of statistical package differs from the one stated in the abstract. Please revise to be consistent on the version of the statistical package used. | We appreciate the comment. This was an oversight, and we have corrected the statistical package described in the abstract to read the 25^th^ version of IBM SPSS |
| 14. Line 175-176: “Descriptive data for continuous variables were analyzed using a measure of central tendencies such as means and standard deviation”. I think authors need just to state specifically the summary measures used (e.g Continuous variables were summarized using means and standard deviations). | We appreciate the comment. We have revised the statement to read “Continuous variables were summarized using means and standard deviations.” |
| 15. Line 176-177: “The sociodemographic characteristics, nurses’ knowledge, nurses’ practice, and organization factors were analyzed using frequencies and percentages”. I think it is not clear why frequency and percentages. The reason is because they are categorical variables. Therefore, I would recommend rephrasing to a “categorical variables were summarized using frequencies and percentages”. | We appreciate the comment. We have revised the statement to read “categorical variables were summarized using frequencies and percentages” |
| 16. Line 179: “All variables with a p-value ≤ 0.05 and a 95% confidence level were considered statistically significant.” is not clear. Consider rephrasing to “All variables with p-value<0.05 (strictly less than 0.05 as in the abstract) was considered significant”. There is no need to use both significance level and confidence level as they mean the same. | We appreciate the comment. We have made a revision by removing the confidence level and restating the *p*-value to strictly less than 0.05. |
| 17. Table 4: Indicate the reference category. | We appreciate the comment. The reference category has been indicated in table 4 |
|  |  |
| **Reviewer #2** |  |
| **Title:** |  |
| It is a good title that assess the HCWs in the health facilities and support the different stakeholders.  Generally, it is good. However, the following comments/questions need to be considered before publication of this manuscript. | We appreciate the comment |
| **Abstracts:** |  |
| You include all sub-section of abstract, which is good. It is better if you remove less important finding from results subsection, for better attraction of readers.  -‘’ Of the 208 respondents, 120(57.7%) were female, 144(69.2%) were in the age group of 20-40, and 175(84.1%) worked in the wards”  - ‘’ None of the socio-demographic (including knowledge of oxygen administration)  and organizational factors were associated with practice on oxygen administration” | We agree with the comment. Less important finding from results subsection have been removed better attraction of readers. These include the following sentences:  -“Of the 208 respondents, 120(57.7%) were female, 144(69.2%) were in the age group of 20-40, and 175(84.1%) worked in the wards”  - “None of the socio-demographic (including knowledge of oxygen administration)  and organizational factors were associated with practice on oxygen administration” |
| **Materials and Methods:** |  |
| - You start with study design. It is better if begin from study setting since most of the articles are like that. | We agree with the comment, we have begun with study settings followed by study design |
| - You didn’t mention your study period. | We appreciate the comment. We have included the study period in the Data collection procedure section |
| - You specifically mention study population, again it is advisable to replace by population/participants characteristics since it include study criteria. | We agree with the comment. We have replaced study population with population characteristics |
| - Under data management and analysis, which type of SPSS Version you have been used specifically? You wrote two version, be consistent. 23/25? | We agree with the comment. It was an oversight and we have corrected the abstract to show we used the 25^th^ version of SPSS software |
| - What cut of point you have been used during bivariate logistic regression to select the candidate of multivariate? | We appreciate the comment. We have included a *p* value cut off point of ≤ 0.2 during bivariate logistic regression to select the candidates for multivariate analysis |
| - You didn’t mention nothing about your data entry mechanism? Model fitness? | Data entry mechanism and the Hosmer-Lemeshow goodness-of-fit test to assess the fit of the logistic regression models to the data have been included |
| **Results:** |  |
| - What was the mean age of your respondents? | The mean age (SD) of our respondents was 36.0 (9.2) and has been included in the sociodemographic characteristics of the respondents in the results section |
| - Table 3? It is not clear for the readers? Try to modify as per standard of regression table. Knowledge status (Good vs Poor; No of/frequency of single among good knowledge vs poor knowledge)? How some body know whether your COR calculation is correct or not? It is better if you modify it. | Table 3 has been revised to include both knowledge status (poor and good) and their frequencies |
| - Table 4 ? How you identify candidate variables ? by p-value <0.2/from previous articles/scientific consideration? Did you consider the reason why not statistically significant but clinically significant? | We appreciate the comment. We have revised allow variables from the bivariate logistic regression with *p* values ≤ 0.2 to be included in the multiple logistic regression model. The reason for not statistically significant but clinically significant is considered and included in the discussion. |
| Discussion |  |
| - What is your standard reference to say lower or higher proportion? I mean you didn’t incorporate CI for your both knowledge and practice? Include it. | We appreciate the concern. We have revised our statement to avoid the use of ‘lower or higher proportion’. Instead, other more relevant specific terms have been used. Moreover, the CI have been included for both knowledge and practice in the tables 3 and 4 reporting logistic regressions. |
