# Supplementary material for: Factors associated with knowledge and practice regarding oxygen administration: A cross-sectional study among registered nurses working in wards and ICUs at Muhimbili National Hospital in Dar es Salaam, Tanzania
Source: PLoS One. 2025 Jan 16;20(1):e0317620. doi: 10.1371/journal.pone.0317620 (PMC11737716; doi:10.1371/journal.pone.0317620)
Supplement: Attachment — Submitted filename: Re-review comment docx.docx [file pone.0317620.s004.docx]

**Factors associated with knowledge and practice regarding oxygen administration: A cross-sectional study among registered nurses at Muhimbili National Hospital in Dar es Salaam, Tanzania**

Journal: PLOS ONE

Manuscript ID: PONE-D-23-37459R1

Manuscript Type: Original Research article

**Reviewers comments/suggestion,**

**Dear  Editors,**

Thank you for your invitation to this manuscript re-review. valuable suggestions, comments, and questions regarding this manuscript. The author needs to consider the following issues before publication, and it is in the author's hands to modify and react to the raised concern. Again, it needs **major modification** before the next step. I have included the comments and suggestions in the table below.

Table 1: Reviewers comments or questions for the authors.

| **N.o** | **Comments/questions** | **Author responses** |
| --- | --- | --- |
| 1. | General comments regarding title modification It is better if you modify it as follows: ‘’**Knowledge and practice of oxygen administration and associated factors among registered nurses at MNH in Dares Salaam, Tanzania: A cross-sectional study**’’ Most KP studies in most literature were written like that. Again, the readers easily capture the “WH” question and answers as well as the nature of your study design. |  |
|  | **Abstracts** |  |
| 2. | Background: rewrite your objectives by incorporating knowledge and practice. Check your title. |  |
| 3. | Methods:   1. Modify a descriptive cross-sectional study into “hospital-based cross-sectional” since it is obvious that you are conducting a descriptive study. 2. “Data were gathered in May 2022." May 1-5,5-10,25-30? 3. “Questionnaire tested by authors: why? 4. “Mean KP scores: this part must be removed from here as per the previous comment.   “Regression analyses were performed to evaluate socio-demographic factors.” You limit your IVs to only SD. Please rewrite this part. |  |
| 4. | “None of the factors showed a statistically significant association,” which is not significant enough to mention here. It looks like part of a discussion. |  |
| 5. | Conclusions:  Your conclusion is a direct copy (of none of the factors) of your result.   1. Rewrite other ways of drawing conclusions based on your 2. What is your baseline for your conclusion that nurses had a poor KP regarding oxygen administration? in relation to what?  national data or international trends, specify it. 3. Rewrite your conclusion. What makes your findings unique from other scientific investigations conducted by scholars? |  |
| 6. | Keywords: put in alphabetical order and Add ‘Daresalam’ |  |
|  | **Introductions** |  |
| 7. | Paragraph 2  Line 52: Such expertise needs citation.  Line 59: Remove the full stop before the closed parenthesis. |  |
| 8. | 1. Write the burden caused by the problem clearly in this part. 2. You are mixing the magnitude of practice and associated factors from lines 64 to 69. You have to separate them. 3. The same is true for knowledge. line 71-78. |  |
| 9. | What are the interventions taken by WHO ? Add them. |  |
| 10. | Paragraph 5, line 83-84, cross check along with your titles. |  |

|  | **Methods and Materials** |  |
| --- | --- | --- |
| 1. | This part need a lot of re arrangement |  |
| 2. | Under your study design, “This approach was considered appropriate to quantify the factors.” Why is it important to justify your design? Are you sure the cross-section is better than the others to identify associated factors? |  |
| 3 | Please merge “study design, period and area” and write it into 2 paragraph. |  |
| 4 | Your method part is not well organized.  Next, sample size and sampling procedure:  Put: Data collection tool and procedure Who will be your data collectors? (about the qualification of a research assistant or supervisor)  Study variables (put separately)  measurement or operational definition  Data quality control  Data processing and analysis |  |

|  | **Results** |  |
| --- | --- | --- |
| 1. | Under socio-demographic par:  Line 207 is not clear. Mean age: …36 ()  Rewrite it as “the mean age (±SD) of the mothers was 36 (±9.2) years.” |  |
| 2. | Line 216 need clarification like the above. |  |
| 3. | Under factors associated with nurses knowledge of O2 administration:  Bivariable LR is the stage at which we select a candidate variable for multivariable LR. When did we say the outcome variable was statistically significant? |  |
| 4. | Table 2: It is better if you remove bivariate and multivariable LR from this as the previous comment is not addressed. Why did you add a p-value for bivariable LR? Justify it.  For variables such as age, sex, and marital status, nothing is written about the AOR, CI, or p-value. How can we know whether these variables are statistically significant or not?  add their value to this table. |  |
| 5. | Table 3  Age,sex, marital status……AOR,CI and p-value |  |
|  | **Discussion** |  |
| 6. | General comments: You didn’t address all your objectives in this discussion. The objectives, like knowledge and practice, were not discussed. (Even your associated factors in both knowledge and practice are only one.) How can we say your discussion is adequate? |  |
| 7. | Begin your discussion by stating the main problem of your study, its gap, and the intended solution. |  |
| 8. | Your objectives or title include the knowledge and practice parts. Nothing is discussed about them.. |  |
| 9. | What could you conclude fr om your study? |  |
