# Supplementary material for: Factors associated with knowledge and practice regarding oxygen administration: A cross-sectional study among registered nurses working in wards and ICUs at Muhimbili National Hospital in Dar es Salaam, Tanzania
Source: PLoS One. 2025 Jan 16;20(1):e0317620. doi: 10.1371/journal.pone.0317620 (PMC11737716; doi:10.1371/journal.pone.0317620)
Supplement: Attachment — Submitted filename: Response to Reviewers.docx [file pone.0317620.s005.docx]

**Factors associated with knowledge and practice regarding oxygen administration: A cross-sectional study among registered nurses in Dar es Salaam, Tanzania**

**Response to reviewer’s comments**

We would like to thank the reviewer for the comments provided to improve this study. We have revised the manuscript as shown in the point-to-point responses to reviewer’s comments provided in the table below.

| **Reviewers Comments** | **Authors Response** |
| --- | --- |
|  |  |
| **Editor** |  |
| The manuscript still needs further major revision; intensively address all the comments raised by the reviewers. | Thank you for the comment. We have addressed all comments by point to point responses. Some of the comments raised by Reviewer #3 were addressed in the previous revisions that we submitted. Nevertheless, we have addressed all the comments raised at the moment. |
|  |  |
| **Reviewer #3** |  |
|  |  |
| **Title** |  |
| It is interesting and relevant to the field that will be input for literature for future researchers. But study population looks general. Thus, it is a good idea to make it specific like nursing working at ED, ICU, ward and etc. | We appreciate the comment. We accept is and have revised the title and made it specific by adding the suggested terms. It now reads ‘Knowledge and practice of oxygen administration and associated factors among registered nurses working in wards and ICUs: A cross-sectional study at Muhimbili National Hospital in Dares Salaam, Tanzania |
| **Abstract:** |  |
| Well written summarizing what was done and what was found. However, its result part regarding associations should described as positively or negatively associated. Additionally, I will suggest you refer back whether it is academically appropriate to say poor knowledge rather than low/high knowledge on something. | We accept the comment. We have revised the result part of the abstract to describe associations as positively or negatively associated. Additionally, we have replaced poor/good knowledge with low/high knowledge. |
| **Introduction:** |  |
| It clearly summarizes the current state of the topic and addresses the limitations of current knowledge in this field. Additionally, it clearly explains why the study was necessary. | Thank you for the comment |
| **Material and methods:** |  |
| The study design and methods appropriate for the research question. The timeframe of the study sufficient is to see outcomes. It is clear how samples were collected or how participants were recruited from each unit. | We appreciate the comment |
| I am curious what was the sampling frame that authors used for simple random sampling technique and why authors didn’t take 360 nurses as it was feasible. | Thank you for the comment. The same comment was raised by the editor in the first round of review and we addressed it in the last revisions that we submitted. This information was incorrect and therefore was revised and does not appear in the current version of the manuscript. |
| Additionally, I have found one concern under measurements section, authors used mean score to classify knowledge as poor when score is less than mean and good when score greater than is mean. What about those who scored exactly mean? Similar question for practice? | We appreciate the comment. It was an oversight and we have corrected it. We used mean score to classify knowledge as poor when score is less than mean and good when score is equal or greater than the mean. The same correction has been made for practice |
| **Results:** |  |
| The results are presented clearly and accurately, and all the relevant data have been included. But the data described in the text inconsistent with the data in the tables 2 where o2 admin knowledge and practice were described under the Table2 heading “nurses’ Socio-demographic characteristics.” | We appreciate this comment. Again, this comment was addressed in the last revisions we made as raised by the editor. Therefore, o2 admin knowledge and practice have been removed from Table 2. |
| **Discussion:** |  |
| I applaud that author logically explained the findings and compared the findings with current findings in the research field and discussed contradictory data. However, sample size difference as explanation does not sound thus it is advisable to look other scientific justifications for discrepancies. Additionally, the implications of the findings for future research and potential applications as well as limitations of the study are discussed. | We appreciate the comments. Also, this comment was addressed in the last revisions we made as raised by the editor. Therefore, other scientific justifications for discrepancies rather than sample size have been considered. |
| **Conclusion:** |  |
| Authors provided a clear summary of the main points. | We appreciate the comment |
