# Supplementary material for: Factors associated with knowledge and practice regarding oxygen administration: A cross-sectional study among registered nurses working in wards and ICUs at Muhimbili National Hospital in Dar es Salaam, Tanzania
Source: PLoS One. 2025 Jan 16;20(1):e0317620. doi: 10.1371/journal.pone.0317620 (PMC11737716; doi:10.1371/journal.pone.0317620)
Supplement: Attachment — Submitted filename: Response to Reviewers_2_MI_Joel.docx [file pone.0317620.s006.docx]

**Factors associated with knowledge and practice regarding oxygen administration: A cross-sectional study among registered nurses in Dar es Salaam, Tanzania**

**Response to reviewer’s comments**

We would like to thank the reviewer for the comments provided to improve this study. We have revised the manuscript as shown in the point-to-point responses to reviewer’s comments provided in the table below.

| **Reviewers Comments** | **Authors Response** |
| --- | --- |
|  |  |
| **Reviewer #4:** |  |
| **General comment** |  |
| This manuscript tried to explore Nurses’ knowledge and practices of oxygen administration in a referral hospital. The authors also examined the factors associated with these two outcomes. The authors did a good job of identifying this very critical aspect of healthcare that has not received a lot of attention. The manuscript is very well written, except for some minor corrections in the methods and results section. | Thank you. Well noted |
| **Abstract** – Well written | Thank you |
| **Introduction** – Well written | Thank you |
| **Methods** |  |
| Lines 122 to 125  The authors described the respondent selection process as “This technique ensured an equal chance of selecting the respondents. During the selection process, two pieces of paper marked as YES and NO were folded and placed in a box. Potential respondents were asked to pick one paper at a time without repeating. Those who selected a paper marked YES were included in the study.”  If only 2 pieces of paper were what respondents had to select from, then the authors did not ensure that each respondent had an equal chance of being selected. Please, state how many nurses were left after implementing the exclusion criteria and how each respondent was selected based on the principles of random probability if that is what was done. | Thank you for the comment.  The changes have been made in the revised manuscript by including the sampling frame (Line 137-153), page 7 |
| Lines 141 to 146. This statement is not clear. Kindly consider rephrasing. | Thank you. The statements have been rephrased as suggested by the reviewer (Lines 163-164). Page 8 |
| Line 153. Kindly consider replacing ‘another’ with ‘a secondary’ | Thank you. The word “another” has been replaced with “secondary” as suggested |
| Line 161. Kindly correct ‘in’ |  |
| Lines 181 to 183. Kindly provide the Cronbach alpha value for the reliability test. | Thank you for the comment. This has been included in the revised manuscript (line 206-207) |
| Line 195. Indicate the level of confidence used. | Thank you for the comment. A 95% Confidence interval has been included in the revised manuscript. (Line 219-220) |
| **Results** |  |
| Table 2. Write all abbreviations in full or clearly define them in the footnote, including ICU etc | All terms have been written in full as suggested. |
| Table 3. Correct ‘O2’ to O with a subscript 2 | Thank you. O2 has been changed to O^2^ as suggested. Now it is in table 4 |
| **Discussion** – Well written |  |
| Wrongly spelt words: There are a few words that need to be corrected in this manuscript. Check ‘including’ on line 281 and others | Thank you. Grammatical errors have been checked throughout the manuscript, including the suggested line |
| Thank you for the opportunity and good luck to the Authors. | Thank you |
| **Reviewer #5:** |  |
| In general, the topic is required while the manuscript require major revisions.  The manuscript requires English revision and editing using professional editing tools | Thank you. We used the Grammarly online editing tool and assistance from an independent researcher to enhance the language and clarity of the text. |
| **Abstract:** |  |
| - Abstract does not give readers practical implications from your study | Thank you. We have added information to the conclusion of the abstract to address practical implications of our study (Line 387-392) |
| - No need for the following statement “Data were then entered into an excel sheet and 25 transferred into the 25th version of IBM SPSS software for analysis” in the method | Thank you, the statement has been deleted as suggested by the reviewer |
| - What about the instrument used in data collection? This should be reflected in the method | The instrument used in data collection has been described in the methods section. See line 157 to 168 |
| **Introduction:** |  |
| - Background is very weak. Discuss the context of Tanzania in the light of international studies | Thank you. We have added information regarding the context of Tanzania in the light of international studies |
| - Develop a through literature review for your topic | Thank you. We have added more literature to our topic |
| - Generate research gap clearly and in depth, give the reader why you select oxygen administration to test with in your context. | We have revised the last paragraph of the introduction to show the gap clearly and in depth in the Tanzanian context |
| - Discuss the significance of your study | Thank you. We have added some information on the significance of the study |
| - Use thought provoking statements that keep attention of readers | Thank you. We have revised the section to use thoughts provoking statements |
| - You clearly state your aim of study in separate title | Thank you |
| **Method:** |  |
| - Sampling is questionable. Give details about sampling selection and parameters of estimating sample size. Please revise sampling approach as what you mentioned is not random sampling | Thank you for the comment. We have revised the sampling and provided more information to clarify how it was done. |
| - discuss clearly how you develop the study questionnaire. You develop new tool this go through certain steps. I recommend using devellis’ model for scale development otherwise, your study seems that you develop a test for students then you conclude results. | Thank you for your comment. This was a writing error on our part. We adapted questions from standard questionnaires used in previous studies, and we have revised the methods section to accurately reflect this |
| - What about the index of content validity of the newly developed tool? | Thank you. We did not develop a new tool as clarified above |
| - What about the construct validity of newly developed tool? This requires exploratory and confirmatory factor analysis. | Thank you. We did not develop a new tool as clarified |
| - Also, The Cronbach's alpha is not enough to ensure reliability of newly developed tools, so you need to conduct test retest or item dimension correlations and interdimensional correlations | Thank you. We did not develop a new tool as clarified |
| - What about common method bias? You need to use Harman’s one-factor test | Thank you. We did not develop a new tool as clarified above |
| - How do you screen respondents’ response bias or misconduct? | Thank you. We included social desirability as one of the limitations of our study and measures taken to mitigate them |
| **Results:** |  |
| - On what base do you build regression? You need to conduct preliminary statistics that ensure if you need regression or not as a nova | Thank you for the comment. We have made a revision and performed a Chi-square test as preliminary statistic on which the multiple logistic regression analysis builds |
| **Discussion:** |  |
| It does not follow guidelines of effective debate | Thank you for the comment. We have revised the discussion to incorporate effective debate |
| Despite you writing lengthy statements about implications, I cannot find what you conclude from your study. What are the clinical implications from your study I see general implications that could be written without research. | Thank you for the comment. We have revised the clinical implications of our study. We hope now that it now conveys relevant information. |
