# Supplementary material for: Factors associated with knowledge and practice regarding oxygen administration: A cross-sectional study among registered nurses working in wards and ICUs at Muhimbili National Hospital in Dar es Salaam, Tanzania
Source: PLoS One. 2025 Jan 16;20(1):e0317620. doi: 10.1371/journal.pone.0317620 (PMC11737716; doi:10.1371/journal.pone.0317620)
Supplement: Attachment — Submitted filename: Response to Reviewers_3_MI_Joel.docx [file pone.0317620.s007.docx]

**Factors associated with knowledge and practice regarding oxygen administration: A cross-sectional study among registered nurses in Dar es Salaam, Tanzania**

**Response to reviewer’s comments**

We would like to thank the reviewer for the comments provided to improve this study. We have revised the manuscript as shown in the point-to-point responses to reviewer’s comments provided in the table below.

| **Reviewers Comments** | **Authors Response** |
| --- | --- |
|  |  |
| **Journal Requirements:** |  |
| Please review your reference list to ensure that it is complete and correct. If you have cited papers that have been retracted, please include the rationale for doing so in the manuscript text, or remove these references and replace them with relevant current references. Any changes to the reference list should be mentioned in the rebuttal letter that accompanies your revised manuscript. If you need to cite a retracted article, indicate the article’s retracted status in the References list and also include a citation and full reference for the retraction notice. | Thank you for the comment. We have gone through the reference list, one by one, and have made a correction to reference number 39 and 45 in the previous manuscript which appeared twice. In addition, we have revised reference number 41 in the previous manuscript which was not complete. |
|  |  |
| **Reviewer #4:** |  |
| The authors have adequately addressed all comments raised by the reviewer. The authors should, however, look through literature for the correct symbol for oxygen. | Thank you for the comment. We have checked in literature and made a revision for the symbol for oxygen to read ‘O_2_’. |
| **Reviewer #5:** |  |
| Despite you making some amendments in this manuscript, the scientific rigor of the study is still questionable.  You should tell how you adapt your tools. How do you ensure the validity and reliability of study tools?  How do you control common method bias? I gave you recommendations that could align this section with scientific principles of research; please see my previous recommendations.  thanks | We appreciate this comment and have revisited the previous comments provided by the reviewer. However, as we indicated in our previous response to reviewers, we did not develop the tools used in our study but adopted them from a previous study in Egypt. We realized that the statement needed to reflect 'adopting' rather than 'adapting' the tools. In the revised manuscript, we have clarified that we adopted the tools and added some details about how their validity and reliability were established by the developers. |
